# Fiske Medical Prize

**Published:** 1857-09

**Authors:** 


*Fiske Medical Prize.*—We notice in the Cincinnati Medical Observer, that the Trustees of the Fiske Fund, at the annual meeting of the Rhode Island Medical Society, held at Providence, June 3, 1857, awarded the premium of $100 to David Hutchinson, M. D., of Moorsville, Morgan county, Indiana.

The following subjects are proposed for 1858: 1st. "The effects of the use of Alcoholic liquors on tubucular disease, or in constitutions predisposed to such disease," to be supported by facts presented as far as possible in a statistical form.

2d. "The morbid effects of retention in the blood of the elements of the urinary secretions.

For the best dissertation on each of these subjects, the trustees will pay $100.

